# Prognostic accuracy of severity grading score and severity scoring index for predicting severe outcomes in Crimean-Congo hemorrhagic fever: a systematic review and meta-analysis

**DOI:** 10.1007/s15010-026-02765-3

**Published:** 2026-03-12

**Authors:** Beatriz Rodríguez-Alonso, Montserrat Alonso-Sardón, Amparo López-Bernus, Ángela Romero-Alegría, Josué Pendones Ulerio, Juan Luis Muñoz Bellido, Antonio Muro, Hugo Almeida, Moncef Belhassen-García

**Affiliations:** 1 Servicio de Medicina Interna, Unidad de Enfermedades Infecciosas, Hospital Universitario de Salamanca (HUS), Centro de Investigación de Enfermedades Tropicales de la Universidad de Salamanca (CIETUS), Instituto de Investigación Biomédica de Salamanca (IBSAL), Universidad de Salamanca (USAL), Salamanca, Spain; 2 Área de Medicina Preventiva, Epidemiología y Salud Pública, Facultad de Medicina, Centro de Investigación de Enfermedades Tropicales de la Universidad de Salamanca (CIETUS), Instituto de Investigación Biomédica de Salamanca (IBSAL), Universidad de Salamanca (USAL), Salamanca, Spain; 3 Servicio de Medicina Interna, Unidad de Infecciosas, HUS, IBSAL, CIETUS, Universidad de Salamanca, Salamanca, Spain; 4 Servicio de Medicina Interna, Unidad de Enfermedades Infecciosas, Hospital Universitario de Salamanca (HUS), Centro de Investigación de Enfermedades Tropicales de la Universidad de Salamanca (CIETUS), Grupo Enfermedades Infecciosas y Tropicales (E-INTRO), Instituto de Investigación Biomédica de Salamanca (IBSAL), Salamanca, Spain; 5 Servicio de Microbiología y Parasitología, HUS, IBSAL, CIETUS, Universidad de Salamanca, Departamento de Ciencias Biomédicas y del Diagnóstico, Universidad de Salamanca, CSIC, Salamanca, Spain; 6 Enfermedades Infecciosas y Tropicales (e-INTRO), IBSAL, CIETUS, Universidad de Salamanca, Salamanca, Spain

**Keywords:** Crimean-Congo hemorrhagic fever, Prognostic scores, Severity grading score, Severity scoring index, Disease severity, Mortality

## Abstract

**Purpose:**

Early risk stratification is critical in Crimean-Congo hemorrhagic fever. We compared the prognostic accuracy of the Severity Grading Score and the Severity Scoring Index for predicting severe outcomes and mortality in laboratory-confirmed Crimean-Congo hemorrhagic fever virus.

**Methods:**

We conducted a systematic review and meta-analysis of prognostic accuracy studies according to PRISMA 2020 guidelines. MEDLINE, Embase, Scopus and Web of Science were searched from inception to November 7, 2025. Observational studies evaluating Severity Grading Score and/or Severity Scoring Index and reporting extractable prognostic accuracy data were included. Pooled sensitivity, specificity, likelihood ratios and diagnostic odds ratios (DOR) were estimated using bivariate random-effects models at prespecified cutoffs for mortality prediction (Severity Grading Score ≥ 9; Severity Scoring Index ≥10). Risk of bias was assessed using QUADAS-2.

**Results:**

Fourteen studies met inclusion criteria. Ten studies evaluating Severity Grading Score (n = 1521) and four evaluating Severity Scoring Index (n = 478) were included in quantitative synthesis for mortality. Severity Grading Score showed very high specificity (0.99, 95% CI 0.95–1.00) but limited sensitivity (0.47, 95% CI 0.27–0.69), yielding a pooled DOR of 94.8. Severity Scoring Index demonstrated higher sensitivity (0.78, 95% CI 0.63–0.88) with high specificity (0.97, 95% CI 0.91–0.99) and a pooled DOR of 100.7. Using an observed mortality prevalence of ~9%, Severity Grading Score provided strong rule-in capacity, whereas Severity Scoring Index offered superior rule-out performance.

**Conclusions:**

Both scores are clinically useful prognostic tools in Crimean-Congo hemorrhagic fever, but with distinct roles. Severity Grading Score is better suited for confirming high-risk status, while Severity Scoring Index performs better as an early triage tool to identify patients at risk of death. Prospective head-to-head validation with standardized outcomes is warranted to guide their incorporation into clinical triage protocols and management guidelines.

**Supplementary Information:**

The online version contains supplementary material available at 10.1007/s15010-026-02765-3.

## Introduction

Crimean-Congo hemorrhagic fever (CCHF) is a tick-borne viral disease with a wide geographic distribution across Africa, Asia, the Middle East and south-eastern Europe [1, 2]. Clinical presentation is highly heterogeneous, ranging from a self-limited febrile illness to fulminant hemorrhagic shock and multiorgan failure [2, 3]. Case-fatality rates vary substantially between settings and may exceed 30%, largely depending on disease severity at presentation and access to timely supportive care [1, 4]. Consequently, early identification of patients at high risk of clinical deterioration is critical to guide triage decisions, optimize resource allocation and improve outcomes [3].

Several prognostic tools have been developed to support risk stratification in CCHF, among which the Severity Grading Score (SGS) and the Severity Scoring Index (SSI) are the most widely used. Both scores integrate readily available clinical and laboratory parameters to predict disease severity and mortality [5, 6]. SGS incorporates age, laboratory markers of cytolysis, coagulation and hematological involvement, as well as key clinical features such as hemorrhage and organ failure, and classifies patients into low-, intermediate- and high-risk categories. SSI is based on platelet count, activated partial thromboplastin time, fibrinogen levels, bleeding manifestations and somnolence, generating a score that categorizes disease as mild, moderate or severe. Despite their widespread use, reported prognostic performance of SGS and SSI varies considerably across studies and endemic regions, and their relative clinical roles remain uncertain.

To date, systematic reviews of CCHF have mainly addressed epidemiology and case-fatality rates, without providing a quantitative synthesis of the prognostic accuracy of SGS and SSI or a structured comparison of their performance for predicting severe outcomes. This lack of consolidated evidence limits the development of standardized, evidence-based triage strategies in clinical practice [7].

The aim of this systematic review and meta-analysis was to evaluate and compare the prognostic accuracy of SGS and SSI in patients with laboratory-confirmed CCHF, focusing on their ability to predict mortality and severe disease. By clarifying the strengths and limitations of each score, this study seeks to inform more rational and standardized risk stratification strategies in CCHF management.

## Methods

### Study design and registration

We conducted a systematic review and meta-analysis of prognostic accuracy studies evaluating SGS and SSI in patients with CCHF. The review was designed and reported in accordance with the PRISMA 2020 statement and the PRISMA-DTA extension for diagnostic test accuracy studies [8]. The protocol was prospectively registered in PROSPERO (CRD420251165815) prior to study selection and data extraction.

### Research question and eligibility criteria

The research question was formulated using the PICO framework, as recommended by the Cochrane Collaboration [9]: “What is the comparative prognostic accuracy of SGS and SSI for predicting adverse outcomes in patients with laboratory-confirmed CCHF?”. Eligibility criteria were defined accordingly (Online Resource 1, Table S1).

We included studies enrolling patients of any age or sex with laboratory-confirmed CCHF diagnosed by reverse-transcription polymerase chain reaction and/or serology. The index tests of interest were the SGS and SSI, applied as prognostic tools at hospital admission or within the first 48 hours. We extracted the timing of score calculation (at admission vs within ≤ 48 h of presentation) when reported; when unclear, it was recorded as “unclear” and considered a potential source of heterogeneity. Studies using modified or unvalidated versions of these scores were excluded. Eligible studies were required to report, or allow calculation of, at least one measure of prognostic accuracy (sensitivity, specificity, AUC, likelihood ratios or diagnostic odds ratio).

The primary outcome was mortality. Severe disease (e.g., ICU admission, shock, organ failure) was considered a secondary outcome; however, insufficient extractable 2 × 2 data precluded quantitative synthesis for non-fatal severe disease outcomes. Observational cohort, cross-sectional studies and randomized controlled trials (RCTs) were included, while case reports, case series (< 5 patients), reviews, editorials and conference abstracts without sufficient extractable data were excluded. However, no RCTs evaluating SGS/SSI prognostic accuracy with extractable data were identified.

### Information sources and search strategy

We searched MEDLINE/PubMed, Embase, Scopus and Web of Science Core Collection from inception to November 7, 2025, without language or date restrictions. Search terms combined concepts related to CCHF and prognostic severity scores. The full search strategy is provided in Online Resource 1 (Table S2).

### Selection process

Records were screened using Rayyan®. Two reviewers independently assessed titles, abstracts and full texts against eligibility criteria. Disagreements were resolved by consensus or consultation with a third reviewer. Reasons for exclusion at the full-text stage were documented in accordance with PRISMA recommendations.

### Data collection process and data items

Data were independently extracted by two reviewers using a standardized, piloted form. Extracted data included study characteristics, population details, index test characteristics, outcome definitions and prognostic accuracy measures. Discrepancies were resolved by consensus or third-party adjudication.

### Study risk of bias assessment and reporting biases

The risk of bias and concerns regarding applicability of each included study were assessed using the QUADAS-2 tool, adapted to the prognostic context. We evaluated four domains: (i) patient selection; (ii) index test (SGS/SSI); (iii) reference standard (mortality or severe disease outcome); (iv) flow and timing.

Within each domain, signaling questions addressed potential sources of bias such as non-consecutive enrolment, case-control designs, inappropriate exclusions, knowledge of outcomes when interpreting the scores, non-pre-specified thresholds, differential verification, or significant delays between score assessment and outcome evaluation. Each domain was rated as low, high or unclear risk of bias, and concerns regarding applicability to our review question were judged similarly.

### Synthesis methods, effect measures and certainty of evidence

Quantitative meta-analysis was performed when sufficient data were available to support hierarchical modeling. For mortality, meta-analysis was performed when sufficient 2 × 2 data at prespecified thresholds were available. For non-fatal severe disease outcomes, results were synthesized descriptively due to insufficient extractable 2 × 2 data.

For each study, 2 × 2 contingency tables were constructed and sensitivity, specificity, positive and negative likelihood ratios (LR^+^, LR^−^), and diagnostic odds ratios (DOR) were calculated with 95% confidence intervals (Online Resource 1, Table S3).

For the primary mortality analysis, clinically used cutoffs were prespecified to harmonize classification across studies: SGS ≥ 9 versus < 9 and SSI ≥ 10 versus < 10. When multiple thresholds were reported, all were extracted, but only the prespecified cutoffs were included in the primary quantitative synthesis. Studies without sufficient data to reconstruct 2×2 tables at these thresholds were included in the qualitative synthesis but excluded from pooled analyses.

Where appropriate, pooled sensitivity and specificity estimates were obtained using bivariate random-effects models [10], and summary receiver operating characteristic (SROC) curves were generated. Because only four studies evaluated SSI for mortality, HSROC curves and prediction regions were not constructed for SSI; instead, pooled estimates were reported and individual study results were displayed in ROC space alongside the summary operating point. From these models we derived 95% confidence regions and, when supported by the data, 95% prediction regions (expected sensitivity-specificity pairs in a new study), displayed on the HSROC plot. Between-study heterogeneity was assessed using variance components (τ^2^) and visual inspection of SROC plots.

Indirect comparisons between SGS and SSI were performed by comparing pooled operating characteristics, because all included studies evaluated a single score and no head-to-head cohorts were available. Comparative effects were computed on the logit scale for sensitivity and specificity using the pooled logit estimates and their standard errors, assuming independence between pooled estimates because the SGS and SSI evidence sets did not overlap. Relative DOR was derived from pooled DORs on the log scale. No paired (head-to-head) analyses were conducted because no study assessed both scores within the same cohort...

Small-study effects and publication bias were explored using funnel plots of log DOR against its standard error and Egger’s test where appropriate. If evidence of asymmetry was identified, the trim-and-fill method was planned to estimate the potential impact of missing studies. The overall certainty of evidence for each outcome and score was assessed using the GRADE approach adapted for diagnostic and prognostic accuracy studies.

## Results

### Study selection and characteristics

**Fig. 1:**
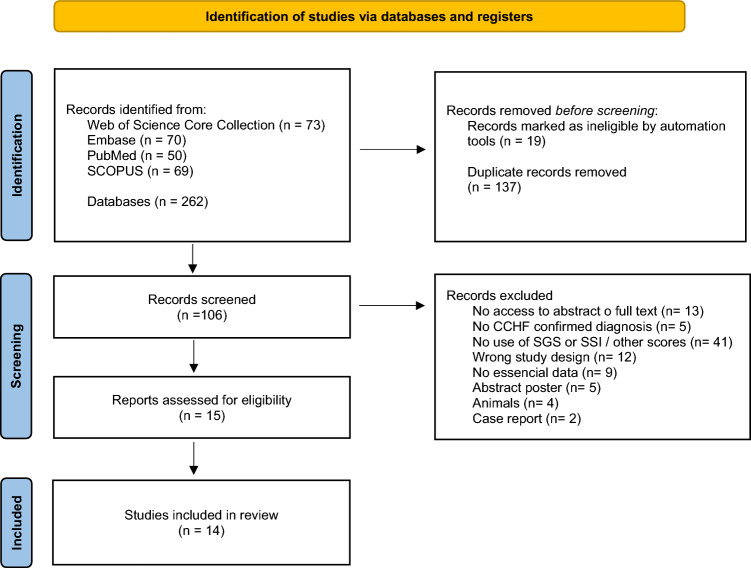
PRISMA 2020 flow diagram (Source: Page MJ et al. BMJ 2021; 10.1136/bmj.n71 [11])

A total of 262 records were identified across the four databases searched. After removal of duplicates and application of eligibility criteria, 14 studies met inclusion criteria and were included in the systematic review. The study selection process is summarized in a PRISMA 2020 flow diagram (Fig. 1).

All eligible studies were included in the qualitative synthesis (Table 1). One report was excluded at the eligibility stage because it did not provide extractable data at the prespecified cutoff (SGS ≥ 9), reporting only lower-threshold classifications (e.g., SGS < 3 and < 4) [12]. Across included studies, score calculation was reported either at admission or within the first 48 hours; however, timing was not consistently reported in all cohorts and may contribute to heterogeneity.

**Table 1:** Study characteristics

Score	Authors (year)	Country	N total	N deaths	Ribavirin	Study_type	Study period (months)	Included in meta-analysis
Severity Grading Score (SGS)	Bakir et al. (2015) [5]	Turkey	404	25	NR	Prospective	5	Yes
	Bakir et al. (2016) [13]	Turkey	72	6	NR	Prospective	4	Yes
	Bozkurt et al. (2016) [14]	Turkey	35	8	NR	Retrospective	12	Yes
	Güler et al. (2016) [15]	Turkey	20	5	0%	Prospective	3	Yes
	Demirtas et al. (2020) [16]	Turkey	97	12	NR	Prospective	9	Yes
	Ersan et al. (2020) [17]	Turkey	60	2	NR	Prospective	NR	Yes
	Bozkurt and Esen (2021) [18]	Turkey	108	21	NR	Retrospective	60	Yes
	Bakir et al (2022) [7]	Turkey	388	16	NR	Prospective	6	Yes
	Ünver-Ulusoy et al. (2022) [19]	Turkey	115	13	0%	Retrospective	79	Yes
	Bozkurt et al. (2025) [20]	Turkey	223	24	NR	Retrospective	110	Yes
Severity Scoring Index (SSI)	Dokuzoguz et al. (2013) [6]	Turkey	281	23	~84%	Retrospective	84	Yes
	Kalın et al. (2014) [21]	Turkey	81	2	~7,4%	Retrospective	36	Yes
	Ergönül et al. (2017) [22]	Turkey	52	8	88%	Retrospective	24	Yes
	Beştepe-Dursun et al. (2021) [23]	Turkey	64	8	0%	Retrospective	111	Yes

*NR* Not reported

Across included studies, crude mortality ranged from 2.5 to 25% (overall ~8%)

For mortality, the meta-analysis included 10 studies evaluating SGS (total n = 1521; deaths n = 132; pooled prevalence 9%) and 4 studies evaluating SSI (total n = 478; deaths n = 41; pooled prevalence 9%). Only studies reporting extractable 2 × 2 data were eligible for quantitative pooling.

### Risk of bias in studies

Risk of bias and applicability concerns were assessed for all 14 included studies using the QUADAS-2 tool across the domains of patient selection, index test, reference standard, and flow and timing (Table 2). However, most studies were retrospective and reporting of patient flow/timing was often incomplete, which may lead to optimistic estimates. In a subset of studies, thresholds were outcome-informed or derived post hoc, potentially inflating prognostic performance (e.g., specificity).

**Table 2:**
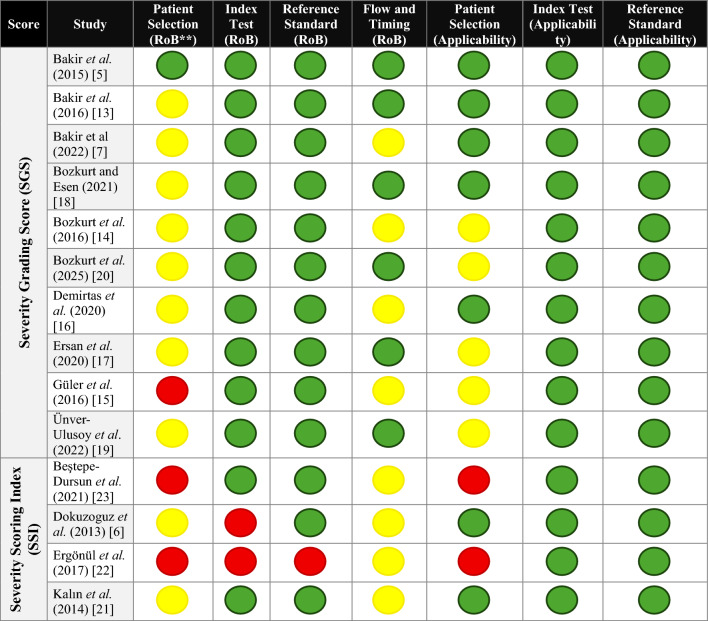
Risk of bias and applicability concerns (QUADAS-2)*

In some studies [6, 22], SSI strata/thresholds were defined using the observed outcome (data-driven or outcome-informed categorization), which may overestimate prognostic performance (e.g., inflate specificity/sensitivity)

^*^Applied a “traffic-light” by study and domain: high/high concern in red, unclear/unclear concern in yellow, low/low concern in green

^**^RoB Risk of bias

Studies (n = 14) showed low risk of bias and high applicability in the reference standard domain, indicating reliable ascertainment of adverse outcomes. The index test domain also demonstrated consistent methodological quality, reflecting appropriate application and interpretation of the SGS and SSI.

In contrast, the flow and timing domain showed greater uncertainty, with seven studies [7, 14–16, 21–23] rated as unclear risk due to insufficient reporting of follow-up duration or timing of outcome assessment. Two studies [15, 23] were judged to be at high risk of bias in the patient selection domain, potentially limiting generalizability. These findings support cautious interpretation of pooled estimates.

### Results of syntheses and certainty of evidence

Using GRADE, the overall certainty of evidence for the prognostic accuracy of SGS and SSI in predicting mortality was rated as low (Online Resource 1, Table S4). Certainty started at low (observational cohorts) and was downgraded for serious risk of bias and indirectness (single-country cohorts), while inconsistency was not considered serious. Imprecision was not serious for SGS and only mildly concerning for SSI. These findings support use as risk-stratification tools, but not for strong context-independent recommendations.

### Pooled prognostic accuracy for mortality

For prediction of mortality, SGS demonstrated moderate pooled sensitivity and very high pooled specificity. Pooled sensitivity was 0.47 (95% CI 0.27–0.69) and pooled specificity was 0.99 (95% CI 0.95–1.00). The pooled diagnostic odds ratio (DOR) was 94.75 (95% CI 23.76–377.79), with a pooled positive likelihood ratio (LR^+^) of 50.31 (95% CI 11.55–219.21) and a pooled negative likelihood ratio (LR^−^) of 0.53 (95% CI 0.35–0.80). Clinically, this indicates that a positive SGS result provides strong evidence for mortality, whereas a negative result only modestly reduces the probability of death. The pooled false-positive rate was 0.01 (95% CI 0.002–0.05).

**Fig. 2:**
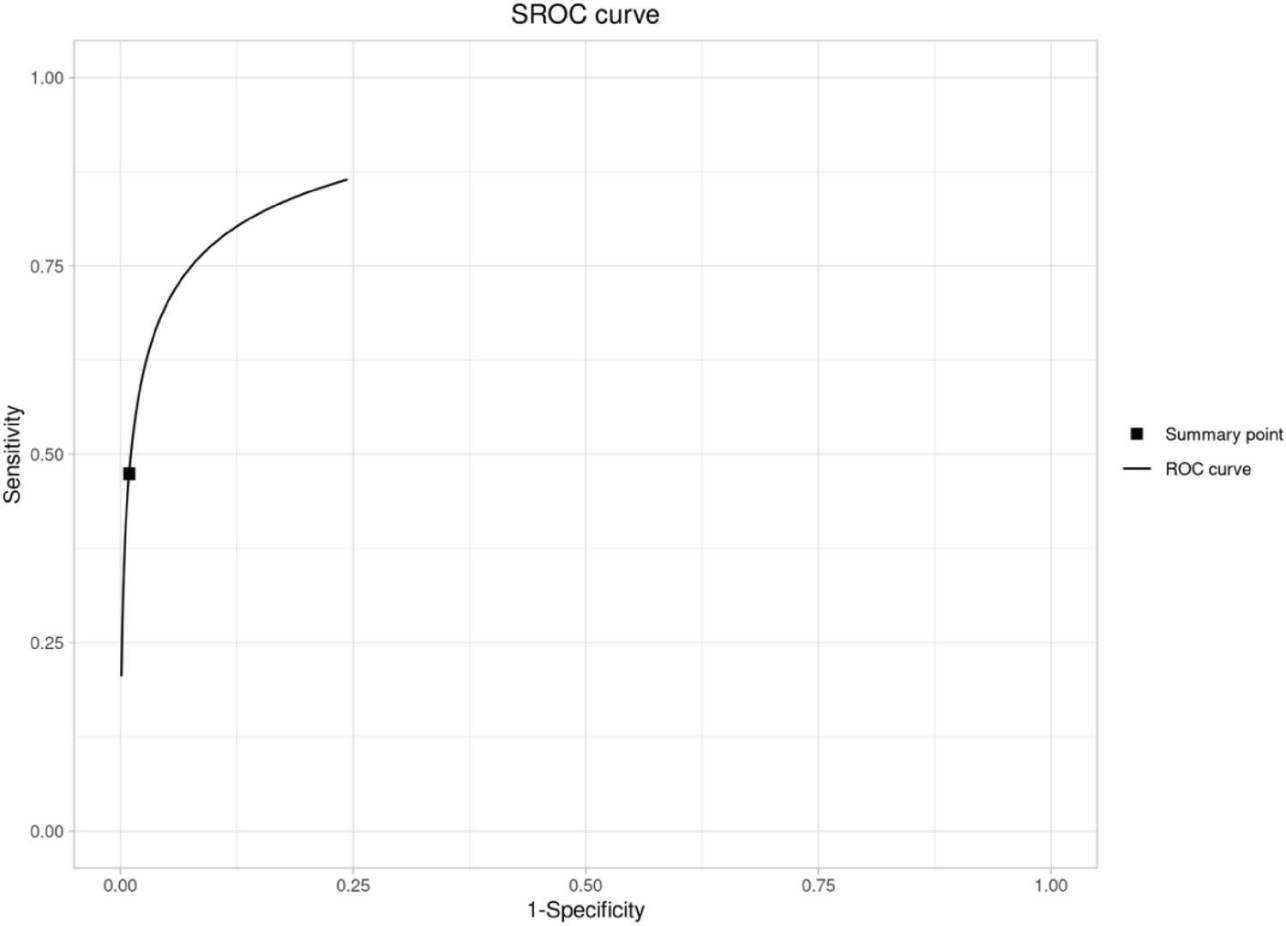
HSROC curve (SGS)

Between-study heterogeneity for SGS was non-trivial, particularly for specificity (bivariate variance on the logit scale: Var logit[sensitivity] = 1.43; Var logit[specificity] = 4.62; bivariate I^2^ = 0.25), indicating clinically relevant variability across studies. An HSROC curve was generated for SGS (Fig. 2). The HSROC plot for SGS includes the 95% prediction region, reflecting the expected range of sensitivity-specificity pairs that may be observed in a future study.

SSI showed higher pooled sensitivity while maintaining high specificity. Pooled sensitivity was 0.78 (95% CI 0.63–0.88) and pooled specificity was 0.97 (95% CI 0.91–0.99). The pooled DOR was 100.71 (95% CI 29.08–348.74), with an LR^+^ of 22.89 (95% CI 8.61–60.83) and an LR^−^ of 0.23 (95% CI 0.13–0.41). From a clinical perspective, the higher sensitivity and lower negative likelihood ratio indicate improved identification of patients at risk of death and a lower probability of missing deaths when the score is negative. The pooled false-positive rate was 0.034 (95% CI 0.01–0.09).

**Fig. 3:**
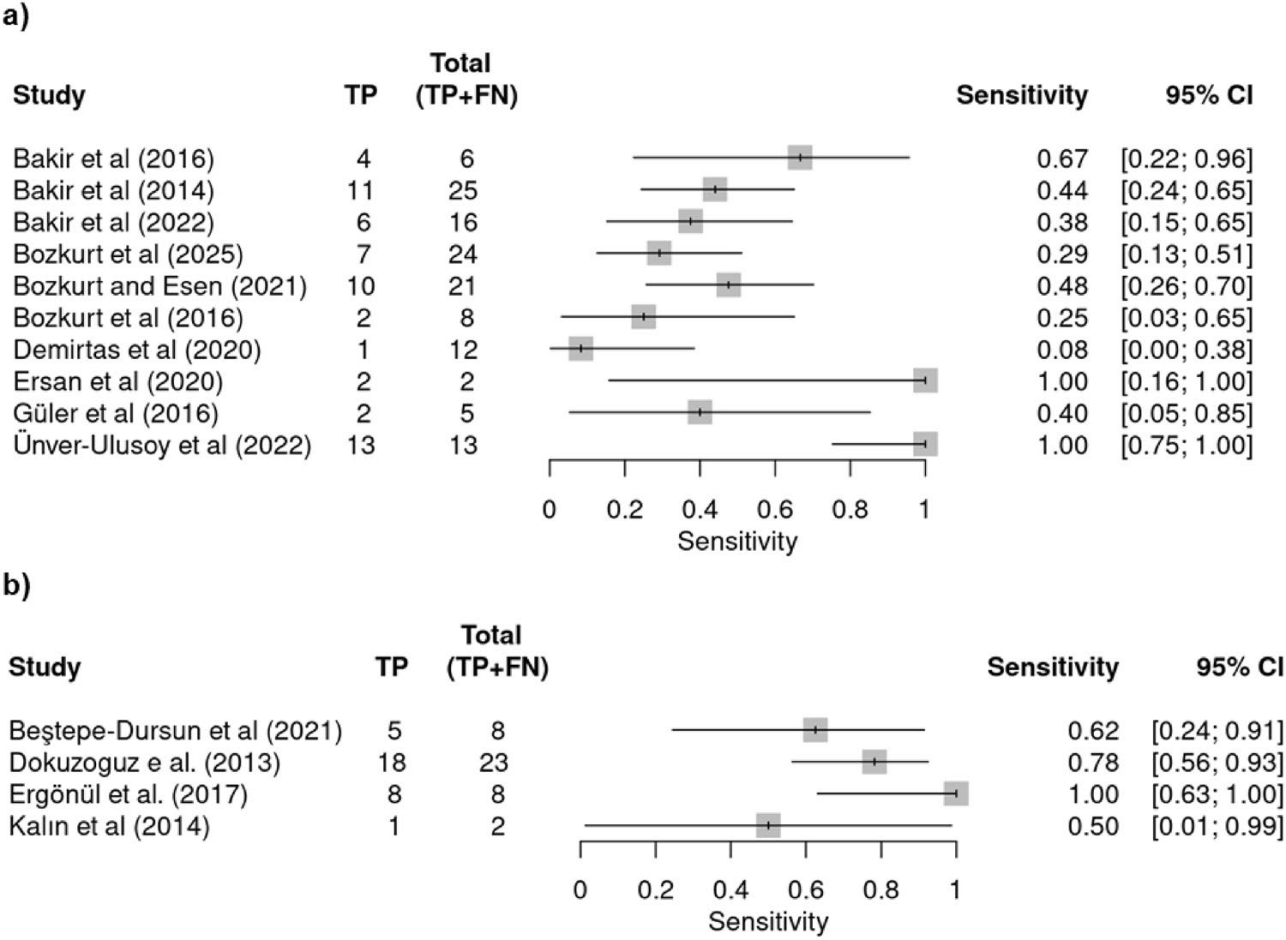
Forest plot of sensitivity: **a** SGS; **b** SSI

**Fig. 4:**
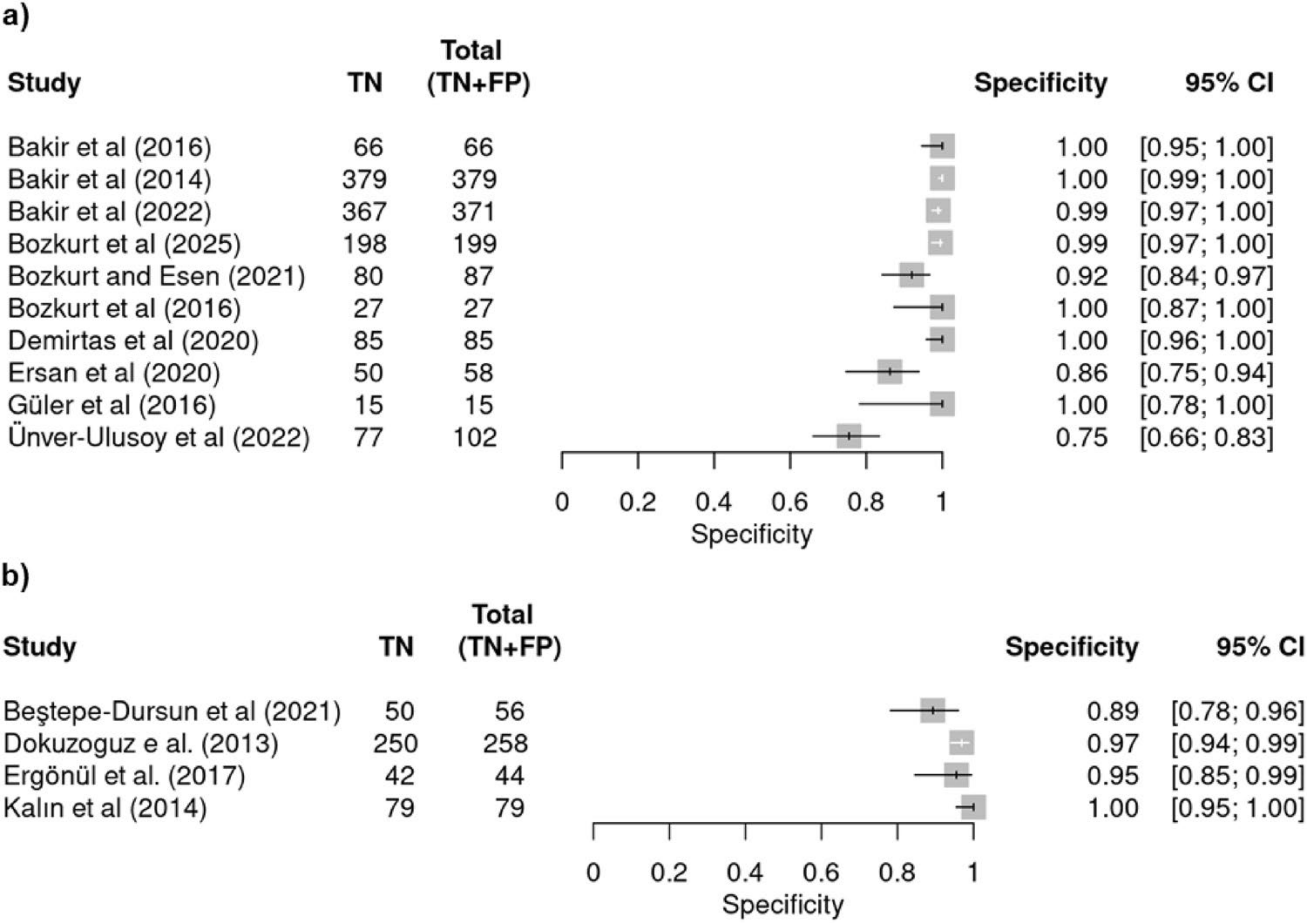
Forest plot of specificity: **a** SGS; **b** SSI

Because only four studies evaluated SSI for mortality, HSROC curves and prediction regions were not constructed. Given the small number of SSI studies (k = 4), prediction regions/intervals were not reported because between-study variance estimation is unstable and may yield unreliable predictions. Instead, pooled estimates and individual study results are presented in ROC space. Study-level sensitivity and specificity estimates for both scores are shown in forest plots (Figs. 3 and 4).

### Clinical interpretation using the observed baseline prevalence

Using the pooled prevalence of mortality (~9%) as a pragmatic baseline risk, a positive SGS result increased the estimated post-test probability of death to approximately 83%, whereas a negative SGS result reduced it to approximately 5%.

For SSI, a positive result increased the estimated probability of death to approximately 69%, while a negative result reduced it to approximately 2%. These post-test probabilities illustrate the complementary clinical profiles of both scores, with SGS providing stronger confirmation of high-risk status and SSI offering greater reassurance when negative. Accordingly, SGS demonstrated stronger rule-in performance, reflected by higher specificity and LR⁺, whereas SSI showed superior rule-out performance due to higher sensitivity and a lower LR⁻. Summary pooled accuracy estimates for both scores are presented in Table 3a.

**Table 3:** Comparative prognostic performance of SGS versus SSI for mortality in CCHF

Metric	SGS* Effect estimates (95% CI)	SSI** Effect estimates (95% CI)
*(a) Pooled accuracy estimates (bivariate random-effects model)*		
Sensitivity	0.47 (0.27–0.68)	0.78 (0.63–0.88)
Specificity	0.99 (0.95-0.99)–)	0.97 (0.91–0.98)
LR+	50.31 (11.54–219.21)	22.89 (8.61–60.83)
LR−	0.53 (0.35–0.80)	0.23 (0.13–0.41)
DOR	94.75 (23.76–377.79)	100.71 (29.08–348.74)

Comparative metric	Effect estimates (95% CI)	*P*-value
*(b) Indirect comparative effects*		
Δlogit (sensitivity) (SSI−SGS)	1.37 (0.22–2.52)	0.01
Δlogit (specificity) (SSI−SGS)	−1.31 (−3.29–0.67)	0.19
Relative DOR (SSI/SGS)	1.06 (0.17–6.82)	0.94

LR+: positive likelihood ratio; LR−: negative likelihood ratio; DOR: diagnostic odds ratio

^*^(k = 10; N = 1521; deaths n = 132; prevalence = 0.09). **(k = 4; N = 478; deaths n = 41; prevalence = 0.09); No paired comparison was possible for mortality because none of the included studies reported both SGS and SSI. Indirect comparative effects were computed on the logit scale for sensitivity/specificity using pooled logit estimates and standard errors; relative DOR was derived from pooled DORs on the log scale. Because the SGS and SSI mortality evidence sets did not overlap, independence between pooled estimates was assumed for the indirect comparisons

A comparative summary of SGS and SSI performance is presented in Table 3b. SGS was more effective for confirming high-risk status when positive, whereas SSI was more suitable for early identification of patients at risk of death.

## Discussion

This systematic review and meta-analysis provide a comprehensive quantitative synthesis of the prognostic performance of the two most widely used CCHF-specific severity scores. Both SGS and SSI demonstrated strong overall discriminatory ability for predicting mortality, as reflected by similarly high pooled diagnostic odds ratios. However, their operating characteristics differed substantially, highlighting distinct and potentially complementary clinical roles [7, 16].

Importantly, the present study was not designed to establish the superiority of one score over the other. Because none of the included studies evaluated both SGS and SSI within the same cohort, all comparative inferences rely on indirect evidence derived from separate study populations. Consequently, our findings should be interpreted as a comparison of operating characteristics rather than as a head-to-head assessment of prognostic performance. This distinction is essential, as indirect comparisons cannot fully account for unmeasured clinical and contextual differences between study settings. Empirical evidence demonstrates that indirect comparisons may yield biased estimates when compared with direct head-to-head evaluations [24, 25], emphasizing the need for prospective comparative studies in the same cohorts.

SGS showed very high specificity but lower sensitivity, supporting its use primarily as a confirmatory (rule-in) tool, whereas SSI showed higher sensitivity while maintaining high specificity, supporting earlier triage and rule-out use. In diagnostic test theory, high specificity with large positive likelihood ratios provides confirmatory evidence when tests are positive, while low negative likelihood ratios improve reassurance when tests are negative [26–28].

These operating differences translate into clinically meaningful shifts in post-test probability across plausible baseline risks (see Results and Table 3), with SGS supporting escalation when positive and SSI supporting de-escalation when negative.

The heterogeneity observed across studies, especially in SGS specificity, likely reflects differences in patient populations, timing of score calculation (admission vs ≤ 48 h), definitions of severe disease and variability in supportive care across endemic and outbreak settings. For SSI, the smaller number of available studies limits the precision of pooled estimates and highlights the need for further validation across diverse healthcare contexts. Nonetheless, the consistency of sensitivity and specificity point estimates across SSI studies supports the robustness of the observed operating profile [29, 30].

Considerable clinical and contextual heterogeneity was observed across included studies, particularly regarding definitions of severe disease, patient case mix, access to intensive care, and supportive management strategies. Rather than undermining the validity of the findings, this variability likely reflects real-world conditions across endemic and outbreak settings. The persistence of distinct operating profiles for SGS and SSI across heterogeneous contexts suggests that the observed differences are more likely related to intrinsic properties of the scores than to setting-specific factors, supporting their generalizability. External validation studies of prognostic models in COVID-19 similarly demonstrated that substantial heterogeneity across settings did not preclude clinical utility when the underlying operating characteristics remained consistent [31].

### Strengths and limitations

This study has several strengths, including the application of hierarchical models for prognostic accuracy and a focus on clinically interpretable measures such as likelihood ratios, which facilitate actionable clinical decisions [10]. Furthermoreour adherence to the PRISMA 2020 statement ensures a transparent and complete reporting of the review process, while the systematic use of QUADAS-2 provides a rigorous appraisal of the potential risks of bias within the available primary literature [10, 26].

Severe disease is clinically relevant for triage; however, non-fatal severe outcomes were heterogeneously defined and rarely reported with extractable 2 × 2 data at prespecified cutoffs. Therefore, our quantitative synthesis focused on mortality, while severe outcomes were summarized descriptively. Nevertheless, important limitations should be acknowledged, including heterogeneity in outcome definitions. In particular, ‘severe disease’ was operationalized using study-specific composite reference standards (e.g., ICU admission, shock, multiorgan failure, transfusion), which may introduce outcome misclassification and indirectness and limited the feasibility and interpretability of quantitative pooling for non-fatal severe outcomes. Importantly, ICU admission and transfusion practices can be influenced by healthcare capacity, local admission thresholds, and referral pathways, such that the composite endpoint may reflect system-level factors in addition to biological severity. Ribavirin use was inconsistently reported and is likely confounded by indication (more severe cases being more likely to receive treatment); therefore, treatment effects cannot be inferred and residual confounding cannot be excluded. Accordingly, we focused the quantitative synthesis on mortality, while severe disease outcomes were summarized descriptively. Additional limitations include limited data for SSI, and the absence of direct head-to-head comparisons of SGS and SSI within the same cohorts, which precluded paired analyses.

The generalizability of our findings is constrained by a significant geographic bias, as nearly all included studies were conducted in Turkey. Consequently, the prognostic accuracy of the SGS and SSI in regions with different viral clades (e.g., Africa), or in healthcare systems with limited ICU capacity, remains uncertain. Furthermore, the lack of data on pediatric populations represents a major evidence gap that precludes any recommendations for this age group.

Furthermore, the included studies exhibited a wide variation in study duration, ranging from 3 to 111 months. This discrepancy suggests a mix of outbreak-driven cohorts, which may capture acute pressures on healthcare systems, and long-term endemic surveillance, which reflects more standardized clinical practice. Such temporal heterogeneity may influence the reported prognostic accuracy, as triage thresholds and resource allocation often shift during the peak of an outbreak compared to periods of stable endemicity.

The risk of bias identified through the QUADAS-2 assessment likely influenced our results. Since many included studies were retrospective, there is a high probability of spectrum bias and post-hoc threshold selection, which typically result in an overestimation of prognostic accuracy. Consequently, the high sensitivities and specificities reported in our meta-analysis should be viewed as an 'upper bound' of performance that requires prospective validation to confirm.

The evidence base for SSI was smaller than for SGS, with only four studies contributing to the quantitative synthesis for mortality [6, 21–23]. Although this limited the precision of pooled estimates and precluded construction of HSROC curves and prediction regions, the consistency of sensitivity and specificity estimates across studies supports the robustness of the observed operating profile. However, further validation in larger, prospective cohorts is warranted to refine precision and confirm these findings [32].

Taken together, these findings support a complementary use of SGS and SSI rather than a competitive interpretation. The concept of complementary diagnostic tools with distinct clinical utilities is well established in other areas of acute care, where sensitive screening tests and specific confirmatory tests are used sequentially to optimize clinical decision-making [33, 34]. SSI appears better suited for early risk stratification and exclusion of death events, whereas SGS may be particularly valuable for confirming high-risk status and prioritizing escalation of care. Prospective head-to-head studies with standardized outcome definitions are now required to formally compare both tools within the same clinical populations.

### Implementation considerations and resource constraints

The implementation of the SGS and SSI in resource-limited settings faces significant barriers. Both scores rely on laboratory parameters that require functional, high-throughput diagnostic facilities. In remote or acute outbreak settings where laboratory turnaround times are prolonged, the window for effective triage may be lost. Besides, the lack of standardized training for frontline staff could lead to inter-observer variability in score calculation, undermining their prognostic utility.

From a public health perspective, the use of these standardized tools allows for a harmonized response across different regions, ensuring that triage decisions are not merely subjective but evidence-based, which is critical in resource-limited settings where CCHF is endemic.

In conclusion, SGS and SSI should not be viewed as competing tools but rather as prognostic scores with distinct clinical utilities. SGS offers excellent rule-in capability for identifying high-risk patients, whereas SSI provides superior sensitivity for early risk stratification. Prospective, multicenter studies with standardized outcome definitions and direct comparative designs are needed to confirm these findings and to inform integration of these tools into clinical decision-making pathways for CCHF.

## Supplementary Information

Below is the link to the electronic supplementary material.

Supplementary file1 (DOCX 56 kb)

Supplementary file2 (DOCX 40 kb)

Supplementary file3 (DOCX 42 kb)

Supplementary file4 (PPTX 189 kb)

## Data Availability

All data extracted are included in the article and supplement file.
