# Supplementary material for: Prognostic accuracy of severity grading score and severity scoring index for predicting severe outcomes in Crimean-Congo hemorrhagic fever: a systematic review and meta-analysis
Source: Infection. 2026 Mar 12;54(3):1253–64. doi: 10.1007/s15010-026-02765-3 (PMC13323525; doi:10.1007/s15010-026-02765-3)
Supplement: Supplementary file 1 — Supplementary file1 (DOCX 56 kb) [file 15010_2026_2765_MOESM1_ESM.docx]

**Online Supplement to**:

**TITLE:** Prognostic accuracy of Severity Grading Score and Severity Scoring Index for predicting severe outcomes in Crimean-Congo Hemorrhagic Fever: A Systematic Review and Meta-Analysis

**JOURNAL**: *Infection*

**AUTHORS AND AFFILIATIONS**

1. **Beatriz Rodríguez-Alonso**, MD, PhD. Servicio de Medicina Interna. Unidad de Enfermedades Infecciosas. Hospital Universitario de Salamanca (HUS). Centro de Investigación de Enfermedades Tropicales de la Universidad de Salamanca (CIETUS). Instituto de Investigación Biomédica de Salamanca (IBSAL). Universidad de Salamanca (USAL). Salamanca, Spain. E-mail: [beamedicina@gmail.com](mailto:beamedicina@gmail.com)
2. **Montserrat Alonso-Sardón**, MD, PhD. Área de Medicina Preventiva, Epidemiología y Salud Pública. Facultad de Medicina, Universidad de Salamanca (USAL). Centro de Investigación de Enfermedades Tropicales de la Universidad de Salamanca (CIETUS). Instituto de Investigación Biomédica de Salamanca (IBSAL). Salamanca, Spain. E-mail: [sardonm@usal.es](mailto:sardonm@usal.es)
3. **Amparo López-Bernus**, MD, PhD. Servicio de Medicina Interna, Unidad de Infecciosas. HUS. IBSAL. CIETUS. Universidad de Salamanca, Salamanca, Spain. Email: [alopezb@saludcastillayleon.es](mailto:alopezb@saludcastillayleon.es)
4. **Ángela Romero-Alegría**, MD, PhD. Servicio de Medicina Interna. Unidad de Enfermedades Infecciosas. Hospital Universitario de Salamanca (HUS). Centro de Investigación de Enfermedades Tropicales de la Universidad de Salamanca (CIETUS). Instituto de Investigación Biomédica de Salamanca (IBSAL). Salamanca, Spain. E-mail: [aralegria@yahoo.es](mailto:aralegria@yahoo.es)
5. **Josué Pendones Ulerio MD, PhD.** Servicio de Microbiología y Parasitología. HUS. IBSAL. CIETUS. Universidad de Salamanca, Departamento de Ciencias Biomédicas y del Diagnóstico, Universidad de Salamanca. CSIC, Salamanca, Spain. Email: [jpendones@saludcastillayleon.es](mailto:jpendones@saludcastillayleon.es)
6. **Juan Luis Muñoz Bellido, MD, PhD** Servicio de Microbiología y Parasitología. HUS. IBSAL. CIETUS. Universidad de Salamanca, Departamento de Ciencias Biomédicas y del Diagnóstico, Universidad de Salamanca. CSIC, Salamanca, Spain. Email: [jlmubel@usal.es](mailto:jlmubel@usal.es)
7. **Antonio Muro, MD, PhD**. Enfermedades infecciosas y tropicales (e-INTRO). IBSAL. CIETUS. Universidad de Salamanca, Salamanca, Spain. Email: [ama@usal.es](mailto:ama@usal.es)
8. **Hugo Almeida*, MD**. Servicio de Medicina Interna. Hospital Universitario de Salamanca (HUS). Centro de Investigación de Enfermedades Tropicales de la Universidad de Salamanca (CIETUS). Instituto de Investigación Biomédica de Salamanca (IBSAL). Universidad de Salamanca (USAL). Salamanca, Spain. E-mail: [hugoalmeida6@gmail.com](mailto:hugoalmeida6@gmail.com).
9. **Moncef Belhassen-García***, **MD, PhD.** Servicio de Medicina Interna. Unidad de Enfermedades Infecciosas. Hospital Universitario de Salamanca (HUS). Centro de Investigación de Enfermedades Tropicales de la Universidad de Salamanca (CIETUS). Instituto de Investigación Biomédica de Salamanca (IBSAL). Email: [belhassen@usal.es](mailto:belhassen@usal.es)

*These authors contributed equally as senior authors of this manuscript.

**Corresponding author**

Moncef Belhassen-García, Servicio de Medicina Interna, Unidad de Infecciosas. CAUSA. IBSAL. Grupo Enfermedades Infecciosas y Tropicales (E-INTRO). CIETUS. Salamanca, Spain.

**Index**:

[Table S1. PICO tool and selection criteria 3](#_Toc223127130)

[Table S2. Search strategy 4](#_Toc223127131)

[Table S3. Extracted 2×2 contingency data by study. 5](#_Toc223127132)

[Table S4. GRADE summary of certainty of evidence for prognostic accuracy of SGS and SSI 6](#_Toc223127133)

# Table S1. PICO tool and selection criteria

| **PICO tool** | | **Inclusion criteria** | **Exclusion criteria** |
| --- | --- | --- | --- |
| **Population** | Patients with laboratory-confirmed diagnosis of CCHF. | - Studies reporting patients of any age or sex with **laboratory-confirmed CCHF**, diagnosed by RT-PCR and/or serology (IgM and/or IgG). Hospital-based cohorts with individual-level clinical data. | - Studies without laboratory confirmation of CCHF; animal studies; community or surveillance reports without individual-level clinical data; case reports or case series with <5 patients. |
| **Intervention** | SGS | - Studies reporting use of the **SGS** applied as a prognostic tool, calculated at hospital admission or within the first 48 hours of diagnosis, using the original or formally validated version of the score. | - Studies using modified, incomplete, or unvalidated versions of SGS; studies assessing only individual components of the score without calculating the total score; scores applied beyond 48 hours from admission. |
| **Comparison** | SSI | - Studies reporting the use of the SSI applied as a prognostic tool under the same conditions (original or validated version, calculated at admission or within 48 hours). | - Studies reporting modified or partial SSI versions; studies not reporting SSI as a complete score; use of other prognostic scores without SGS or SSI. |
| **Outcomes** | - Sensitivity, specificity, DOR, and AUC-ROC for each score. - Compare performance using paired (head-to-head) and indirect analyses. - Explore sources of heterogeneity, such as geographic region, baseline mortality, study design, and ribavirin use, using subgroup analyses, bivariate models, and meta-regression. | - At least one measure of prognostic accuracy for **mortality** (in-hospital or 30-day all-cause death) and/or **severe disease**, defined as ICU admission, hemorrhagic shock, multiorgan failure, or massive transfusion. Outcomes reported with sufficient data to calculate sensitivity, specificity, AUC, LR⁺/LR⁻, DOR, or to reconstruct a 2×2 contingency table. | - Studies not reporting mortality or severe disease outcomes; studies lacking sufficient numerical data to derive prognostic accuracy estimates or reconstruct 2×2 tables; purely descriptive outcome reporting without accuracy measures. |
| **Additional criteria** |  | - Observational cohort (prospective or retrospective) and cross-sectional studies. | - Case reports and small case series with fewer than 5 patients, editorials, letters, expert opinions, narrative reviews and systematic reviews. |

*AUC-ROC: area under the receiver operating characteristic curve; CCHF: Crimean–Congo haemorrhagic fever; DOR: diagnostic odds ratio; ICU: intensive care unit; LR+: positive likelihood ratio; LR−: negative likelihood ratio; SGS: Severity Grading Score; SSI: Severity Scoring Index.*

| Database | Query | Filters |
| --- | --- | --- |
| PubMed / MEDLINE | ("Crimean-Congo Hemorrhagic Fever"[Mesh] OR "Crimean-Congo hemorrhagic fever"[tiab] OR CCHF[tiab]) AND (("Severity Grading Score"[tiab] OR "Severity Scoring Index"[tiab]) OR ((severity[tiab] OR prognos*[tiab] OR predict*[tiab] OR "risk stratification"[tiab]) AND (score*[tiab] OR index[tiab] OR "scoring system"[tiab]))) | *Humans*. |
| Scopus | TITLE-ABS-KEY("crimean-congo hemorrhagic fever" OR cchf) AND (TITLE-ABS-KEY("severity grading score" OR "severity scoring index") OR TITLE-ABS-KEY((severity OR prognos* OR predict* OR "risk stratification") W/3 (score* OR index OR "scoring system") | Document type: *Article*. |
| Web of Science Core Collection | TS=("crimean-congo hemorrhagic fever" OR CCHF) AND (TS=("severity grading score" OR "severity scoring index") OR TS=((severity OR prognos* OR predict* OR "risk stratification") NEAR/3 (score* OR index OR "scoring system") | Document type: *Article*. |
| Embase (Embase.com) | ('crimean-congo hemorrhagic fever'/exp OR 'crimean congo hemorrhagic fever':ti,ab OR cchf:ti,ab) AND ((severity:ti,ab OR prognos*:ti,ab OR predict*:ti,ab OR "risk stratification":ti,ab) AND (score*:ti,ab OR index:ti,ab OR "scoring system":ti,ab OR "clinical prediction rule":ti,ab OR "prognostic model":ti,ab)) AND [humans]/lim | *Humans*. |

# Table S2. Search strategy

# Table S3. Extracted 2×2 contingency data by study.

| **Authors (year)** | **TP** | **FP** | **FN** | **TN** |
| --- | --- | --- | --- | --- |
| **Severity Grading Score (SGS)** | | | | |
| *Bakir et al. (2015)* [5] | 11 | 0 | 14 | 379 |
| *Bakir et al. (2016)* [9] | 4 | 0 | 2 | 66 |
| *Bakir et al (2022)* [7] | 6 | 4 | 10 | 367 |
| *Bozkurt and Esen (2021)* [10] | 10 | 7 | 11 | 80 |
| *Bozkurt et al. (2016)* [11] | 2 | 0 | 6 | 27 |
| *Bozkurt et al. (2025)* [12] | 7 | 1 | 17 | 198 |
| *Demirtas et al (2020)* [13] | 1 | 0 | 11 | 85 |
| *Ersan et al. (2020)* [14] | 2 | 8 | 0 | 50 |
| *Güler et al (2016)* [15] | 2 | 0 | 3 | 15 |
| *Ünver-Ulusoy et al. (2022)* [16] | 13 | 25 | 0 | 77 |
| **Severity Scoring Index (SSI)** | | | | |
| *Beştepe-Dursun et al. (2021)* [17] | 5 | 6 | 3 | 50 |
| *Dokuzoguz et al. (2013)* [18] | 18 | 8 | 5 | 250 |
| *Ergönül et al. (2017)* [19] | 8 | 2 | 0 | 42 |
| *Kalın et al. (2014)* [20] | 1 | 0 | 1 | 79 |

*TP: true positives, FP: false positives, FN: false negatives, TN: true negatives*

# Table S4. GRADE summary of certainty of evidence for prognostic accuracy of SGS and SSI

| GRADE domain | Severity Grading Score (SGS)  (≥9 for mortality) | Severity Scoring Index (SSI)  (≥10 for mortality) |
| --- | --- | --- |
| Study design | Observational cohort studies (all from Turkish endemic centres); initial certainty rated as low. | Observational cohort studies (all from Turkish endemic centres); initial certainty rated as low. |
| Risk of bias | Serious: non-randomised designs, frequent retrospective data collection, limited adjustment for confounding (e.g. ribavirin use, access to intensive care, plasma exchange), and partial incorporation of outcome components into the score. Downgraded 1 level. | Serious: same issues as for SGS (observational design, potential confounding, and overlap between score items and outcome definition). Downgraded 1 level. |
| Inconsistency | Not serious: direction of effect consistent across studies (higher SGS associated with higher risk of death); variability mainly in sensitivity, while specificity is consistently high. | Unclear / probably not serious: all studies show high sensitivity and specificity in the same direction for mortality, but the number of studies is small. |
| Indirectness | Serious: all data derive from Turkish hospitals in endemic regions, with specific referral patterns, supportive care, and baseline risk; generalisability to other regions, outbreak settings, or healthcare systems is uncertain. Downgraded 1 level. | Serious: same concerns; all SSI validations were conducted in Turkish centres, which may limit applicability to different epidemiological and healthcare contexts. Downgraded 1 level. |
| Imprecision | Not serious: pooled estimates based on 10 cohorts (n≈1,500); 95% CIs for specificity and DOR are relatively narrow; sensitivity is moderate but estimated with acceptable precision; conclusions (good rule-in performance) are robust. | Borderline, not downgraded: only 4 cohorts (n≈478), but 95% CIs for sensitivity, specificity and DOR remain reasonably narrow and support a consistent clinical interpretation (better rule-out performance). |
| Publication bias | Suspected: all studies from a single country and a limited number of research groups; no clearly negative validation studies identified; cannot be formally assessed but likely present. | Suspected: same pattern as for SGS (single-country evidence base, no negative studies), suggesting possible publication bias. |
| Upgrading factors | No upgrading: although LR⁺ and DOR are high, large effects may partially reflect context-specific practice and outcome-related variables embedded in the score; residual confounding cannot be excluded. | No upgrading: SSI shows a favourable balance of sensitivity and specificity, but the evidence base is smaller and subject to the same contextual and methodological limitations as SGS. |
| Overall certainty of evidence† | Low (ranging from very low to low across domains).  Evidence supports SGS as a useful complementary tool to “rule in” mortality risk, but with limitations in internal validity and generalisability. | Low (ranging from very low to low).  Evidence supports SSI as a useful complementary tool to “rule out” mortality risk, but based on a small number of studies from a single endemic setting. |

*DOR: diagnostic odds ratio; LR+: positive likelihood ratio; LR−: negative likelihood ratio; SGS: Severity Grading Score; SSI: Severity Scoring Index.*

† Certainty categories per GRADE: high, moderate, low, very low.
