# Supplementary material for: Prognostic accuracy of severity grading score and severity scoring index for predicting severe outcomes in Crimean-Congo hemorrhagic fever: a systematic review and meta-analysis
Source: Infection. 2026 Mar 12;54(3):1253–64. doi: 10.1007/s15010-026-02765-3 (PMC13323525; doi:10.1007/s15010-026-02765-3)
Supplement: Supplementary file 2 — Supplementary file2 (DOCX 40 kb) [file 15010_2026_2765_MOESM2_ESM.docx]

Records removed *before screening*:

Records marked as ineligible by automation tools (n = 19)

Duplicate records removed

(n = 137)

Records screened

(n =106)

Records excluded

No access to abstract o full text (n= 13)

No CCHF confirmed diagnosis (n= 5)

No use of SGS or SSI / other scores (n= 41)

Wrong study design (n= 12)

No essencial data (n= 9)

Abstract poster (n= 5)

Animals (n= 4)

Case report (n= 2)

Reports assessed for eligibility

(n = 15)

Studies included in review

(n = 14)

**Identification of studies via databases and registers**

**Identification**

**Screening**

**Included**

Records identified from:

Web of Science Core Collection (n = 73)

Embase (n = 70)

PubMed (n = 50)

SCOPUS (n = 69)

Databases (n = 262)
