# Supplementary figures and images for: Prognostic accuracy of severity grading score and severity scoring index for predicting severe outcomes in Crimean-Congo hemorrhagic fever: a systematic review and meta-analysis
Source: Infection. 2026 Mar 12;54(3):1253–64. doi: 10.1007/s15010-026-02765-3 (PMC13323525; doi:10.1007/s15010-026-02765-3)

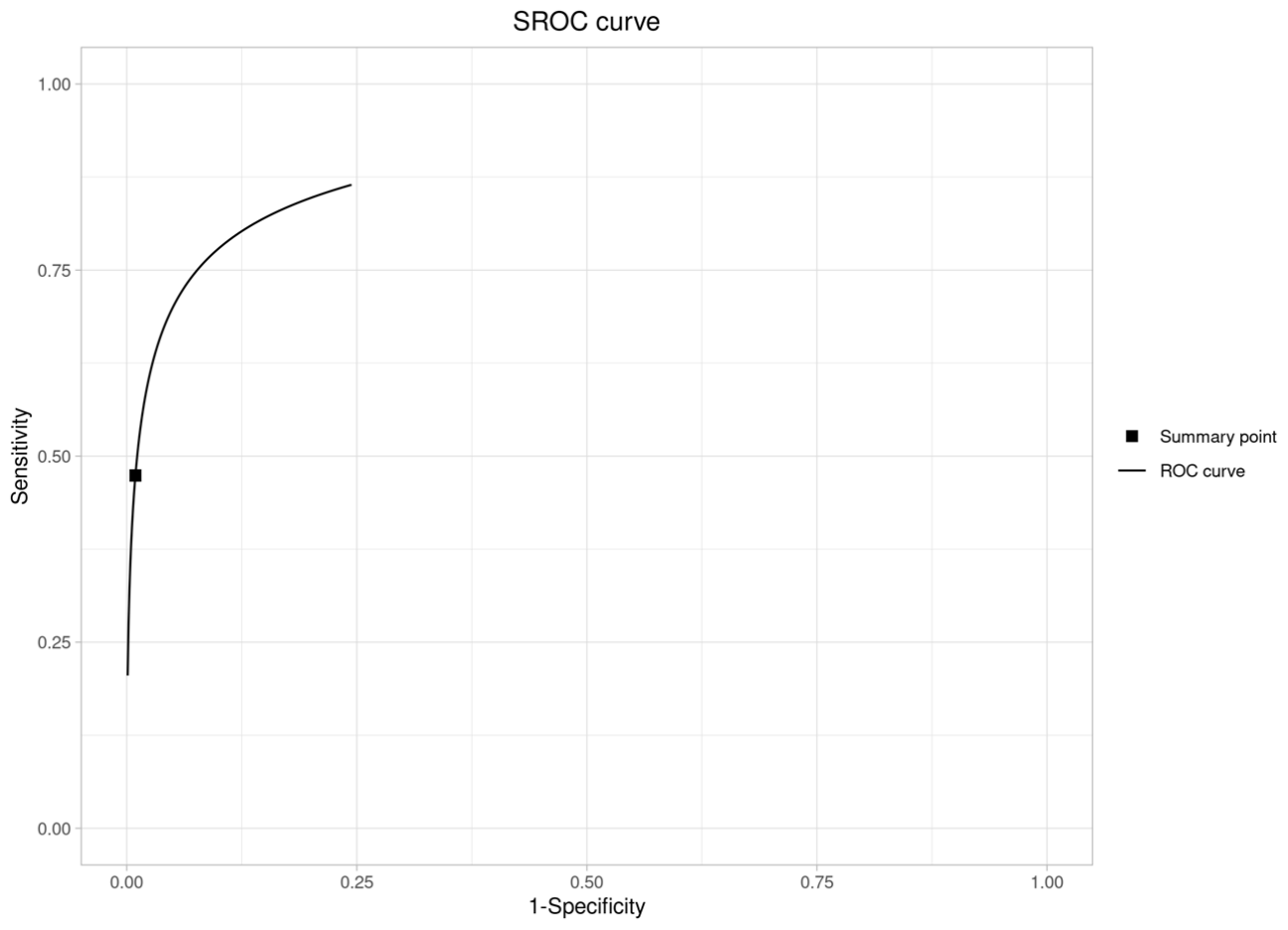

Supplement: Supplementary file 3 — Supplementary file3 (DOCX 42 kb) [file 15010_2026_2765_MOESM3_ESM.docx]

## Slide 1
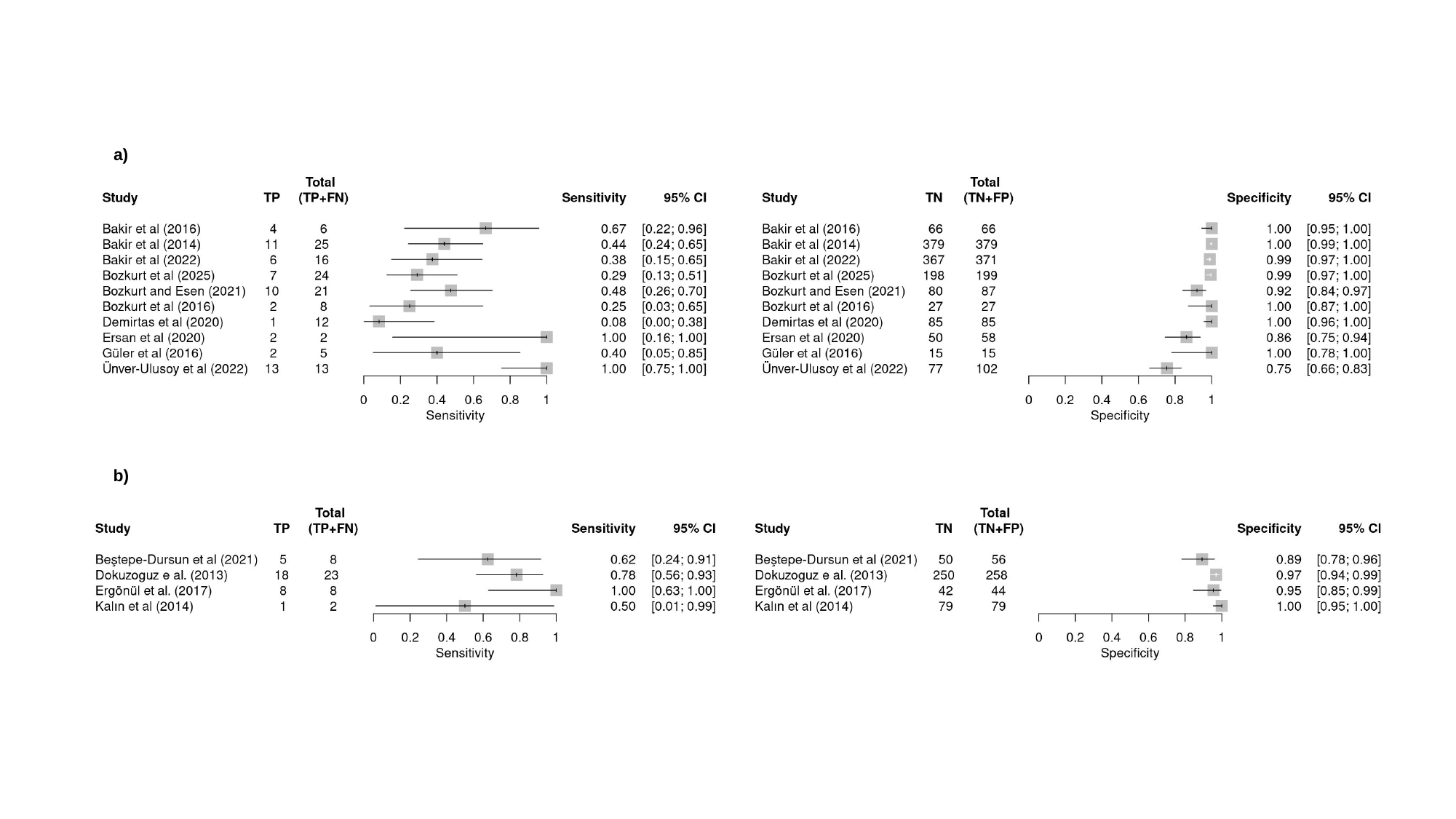

a)
b)

## Slide 2
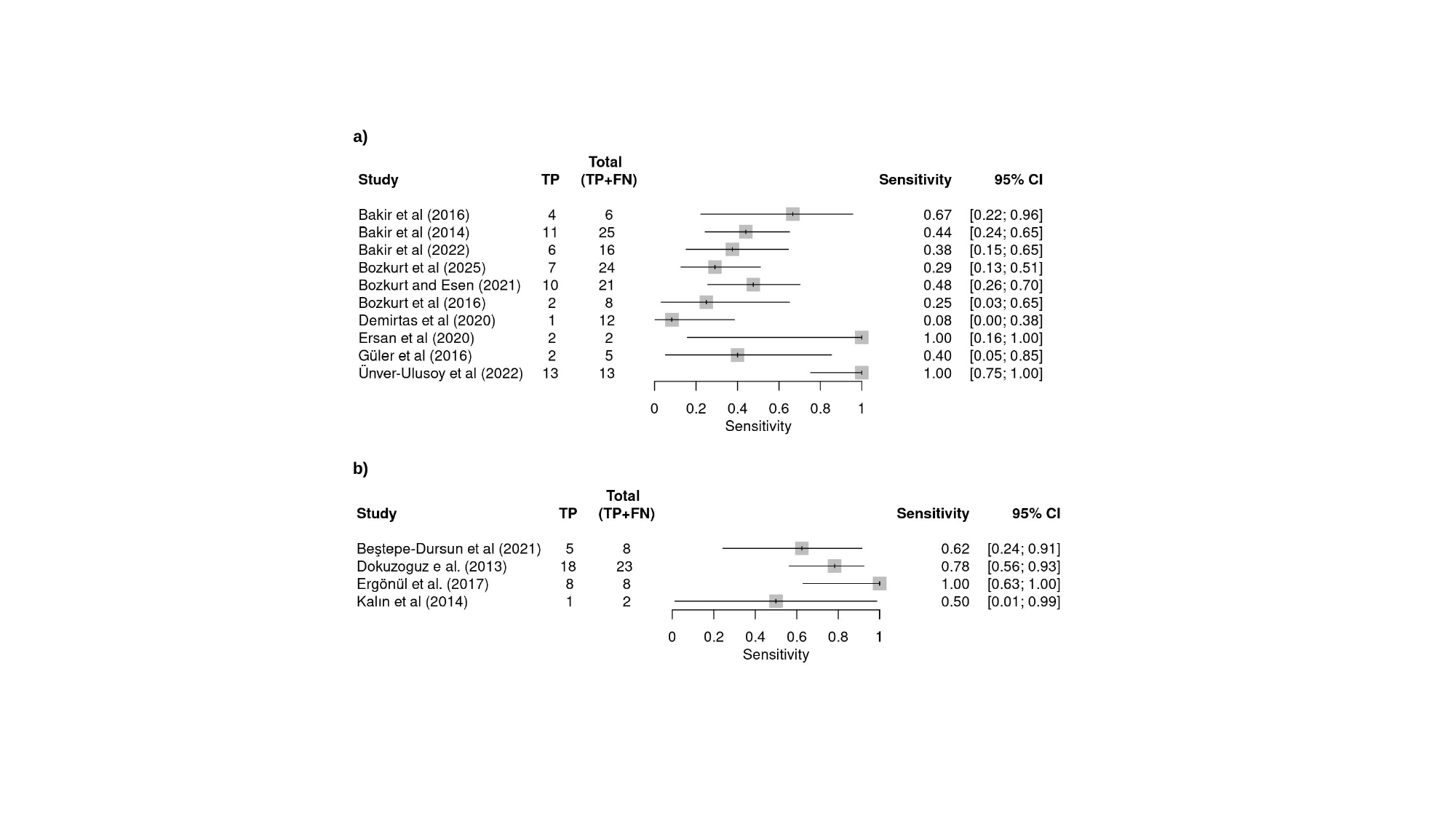

a)
b)

## Slide 3
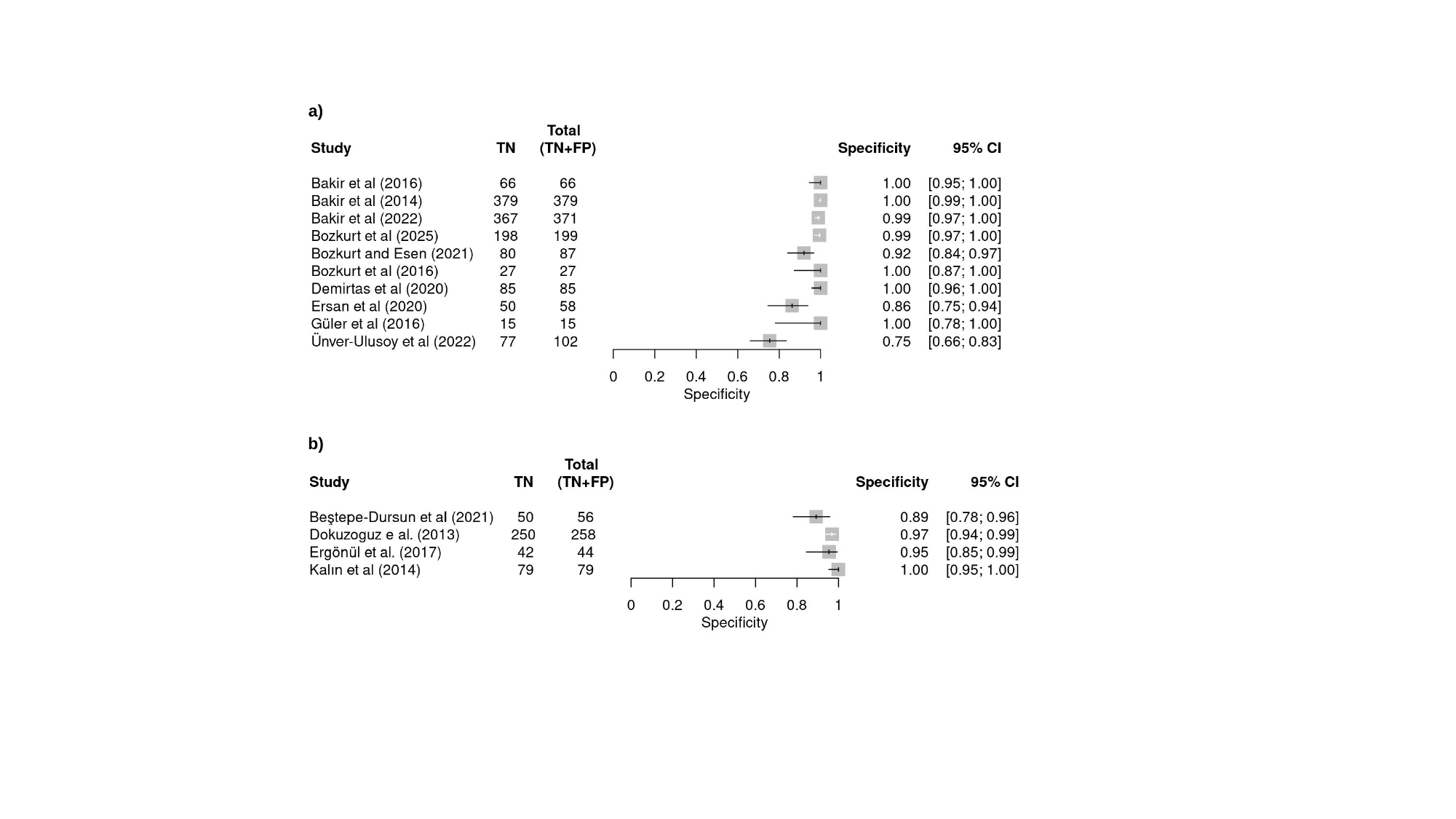

a)
b)

Supplement: Supplementary file 4 — Supplementary file4 (PPTX 189 kb) [file 15010_2026_2765_MOESM4_ESM.pptx]
